# Content-based filter queries on DNA data storage systems

**DOI:** 10.1038/s41598-023-34160-5

**Published:** 2023-04-29

**Authors:** Alex El-Shaikh, Bernhard Seeger

**Affiliations:** Departement of Mathematics and Computer Science, University of Marburg, 35037 Marburg, Germany

**Keywords:** DNA and RNA, Bioinformatics, Software

## Abstract

Recent developments in DNA data storage systems have revealed the great potential to store large amounts of data at a very high density with extremely long persistence and low cost. However, despite recent contributions to robust data encoding, current DNA storage systems offer limited support for random access on DNA storage devices due to restrictive biochemical constraints. Moreover, state-of-the-art approaches do not support content-based filter queries on DNA storage. This paper introduces the first encoding for DNA that enables content-based searches on structured data like relational database tables. We provide the details of the methods for coding and decoding millions of directly accessible data objects on DNA. We evaluate the derived codes on real data sets and verify their robustness.

## Introduction

The amount of data created worldwide is ever-increasing, and the demand for high-density storage systems is growing^1,2^. Though current data storage technologies are constantly improving regarding speed, capacity, and robustness, they are already outpaced and cannot cope with the exponentially growing demand for storage. In addition, current storage devices consume high amounts of energy, are not environmentally friendly, and only persist data for 5–30 years^3^.

DNA-based storage systems exhibit the potential to store an enormous amount of data at very high densities. The estimated theoretical data storage limit of DNA^4^ is $\approx 1$ EB/$\hbox {mm}^3$. Furthermore, DNA is a long molecule found in almost every living cell, encoding the genetic information such as instructions required for survival. The durability of DNA can exceed centuries at operational energy of eight orders of magnitude less than traditional storage media^5,6^. Hence, utilizing DNA as the basis for archival data storage systems seems to be a promising add-on of a green, durable, and dense storage device to today’s storage hierarchy. Recent developments in improving DNA synthesizing and sequencing, as well as the rapid cost declines, further give reasoning to believe that real applications will use DNA storage soon.

So far, current developments in DNA storage systems focus on increasing the amount of data stored^3,7–18^ and successfully restoring the data after reading it from DNA. However, with the ability to store large volumes of data on DNA, it is also crucial to support read requests for only a small portion of the entire data set. This is due to the induced high expenses for naively reading the entire data set compared to just a small portion. Similar to any other storage device, it is cost-beneficial to offer methods for selective data reading.

In this paper, we study the problem of selectively reading data from DNA on tabular data, where a table corresponds to a set of objects with multiple attributes. A table can originate from a relational database or a CSV file. In general, there are two query modes for tables. First, a key-value query retrieves an object using a unique attribute, also known as a key, and returns the associated object (record). Second, an ad-hoc content-based filter query returns the set of objects (records) satisfying a given predicate that is a boolean function of one or multiple attributes. To the best of our knowledge, content-based queries have not been examined for DNA storage so far. Our goal is to design efficient methods solely operating on DNA storage without the need to access other storage devices.

While random access^19^ to DNA is a prerequisite for content-based queries, it does not lead to supporting content-based filter queries without substantial extensions. There are two broad approaches for random access in DNA. The first approach relies on predefined primers for Polymerase Chain Reaction (PCR). A severe deficiency of this approach is that only a very small number of primers are available, leading to a limited number of directly accessible positions in a DNA library. Some techniques mitigate this problem, e.g., by partitioning the DNA library among multiple tubes or using hierarchical primers^10,20^. However, primers are independent of the underlying data and, thus, are generally not suitable for content-based queries.

The second approach supports random access using barcodes as labels for oligonucleotides (oligos). Our previous work^21^ revealed the crucial advantages of using barcodes for managing millions of different objects within a single DNA pool. Furthermore, key-value queries are directly supported by these artificial barcodes. However, similar to primers, these barcodes do not carry any semantics of the data. Therefore, to support key-value queries where the key refers to a unique attribute of the objects, an additional mapping from this key to the barcode has to be managed on disk *in-silico*. For example, consider a DNA library storing a collection of books, where an attribute ISBN unambiguously identifies a book. The basic idea is to store a book object and an associated barcode in an oligo and to store the mapping $\text {ISBN} \mapsto \text {barcode}$ *in-silico*. A key-value query with a given ISBN first transforms the ISBN to the barcode (using the *in-silico* mapping) and then fetches the object from the DNA library using a well-known technique^22–24^. However, there are two deficiencies. First, this approach does not offer a method that uses DNA storage only. Instead, an additional mapping that occupies space, linear in the size of the database, must be kept on a standard storage device. Second, there is no support for content-based queries where a predicate is given on a non-unique attribute. These queries generally return an entire set of objects that fulfill the predicate.

The main contribution of this paper consists of the first method for developing codes that support content-based queries on DNA without the need to keep additional information on an *in-silico* storage device. The key idea is to introduce so-called content-based barcodes (CBBs) that encode information and simultaneously serve as labels for DNA strands. This is an improvement over previous methods, where standard barcodes do not carry any information about the underlying objects. Our DNA is represented by oligos, where each oligo is composed of (i) a CBB, and (ii) an Info-DNA, the part that encodes the data object. Moreover, all oligos are of the length $L_{Oligo}$, which is achieved by padding too short sequences and partitioning too long ones. We present a proof-of-concept of how CBBs facilitate answering ad-hoc content-based filter queries on a database with millions of objects. For example, our method enables retrieving a book by its ISBN (without any mapping) or all books written by a particular author. In order to generate a robust coding of objects (Info-DNA) and their CBBs, we utilize a fountain code, specifically RaptorQ code (RQ)^25^. The resulting DNA codes aim at reducing errors and obeying certain quality constraints. Furthermore, to reduce cross-hybridization noise between DNA sequences, the coding strives to minimize mutual overlaps by approximating the Jaccard similarity with locality-sensitive hashing^26–28^. Experimental results confirm that our approach supports ad-hoc content-based filter queries on collections with up to millions of data objects. Furthermore, Twist Bioscience (https://www.twistbioscience.com/) confirmed the readiness for production of our generated DNA.

## Results

### Overview

**Figure 1:**
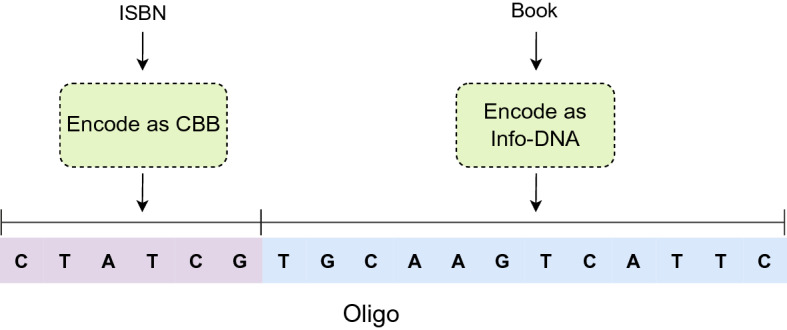
The composition of an oligo in our DNA storage system.

Our encoding scheme is established on the basis of a relational database consisting of tables. A relational table consists of columns (also called attributes) and rows (records). For the sake of simplicity, let Id be the unique attribute, also called the primary key, that identifies a record in a table. This paper exclusively refers to a data object as a record in a relational table. Nevertheless, our encoding methods could also be applied to any kind of digital data. Every data object is encoded as an oligonucleotide (oligo) that is typically a short DNA sequence not longer than 250 base pairs but large enough for storing a record of a table in general. Every oligo in our DNA data storage system is split into two parts, as shown in Fig. 1. Info-DNA encodes the data object, and CBB is a barcode uniquely identifying the corresponding oligo. Moreover, the CBB is used to retrieve the information encoded in the Info-DNA. If the code for a data object is longer than an oligo, the object is split into a chained list of oligos. This is called segmentation. Otherwise, if the data object does not occupy the entire space of an oligo, we fill up (pad) the oligo with artificially generated base pairs to improve the stability, e.g., adjusting the GC content. In the following, we use $L_{Oligo}$ to express the fixed length of an oligo. Furthermore, $L_{CBB}$ and $L_{Info} = L_{Oligo} - L_{CBB}$ denote the length thresholds of CBBs and Info-DNA, respectively. The setting of these two parameters is essential for the error to quantify coding quality and will be detailed in the following sections.

**Figure 2:**
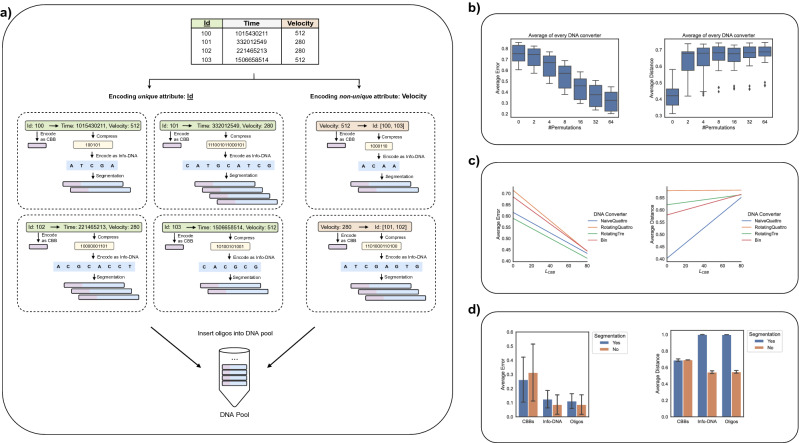
In (**a**), we depict an example of encoding the attributes Id and Velocity of an example table. Id is the primary key. Hence, we encode each value of Id as a CBB, and every CBB is attached as prefix to an Info-DNA. Each Info-DNA encodes the compressed (by GZIP) remaining attributes Time and Velocity of the corresponding record. For example, the values (Time=1015430211, Velocity=512) of the record with (Id=100) are compressed and then encoded with fountain code to an Info-DNA. In our example, this produces (ATCGA) as Info-DNA. If the resulting Info-DNA does not adhere to the constraints C1, C2, C4, and C5, we further apply segmentation. Velocity is not unique and is encoded as follows. First, each unique value is encoded as a CBB, and each CBB is attached to a list of Ids of the associated records. In our example, (Velocity=512) provides the list composed of (Id=100) and (Id=103). The encoding then returns (ACAA). Each list is further compressed and encoded as Info-DNA by the fountain code. We apply segmentation if the resulting Info-DNA violates any constraints C1, C2, C4, and C5. All of the obtained oligos are stored in a single DNA pool. Figures (**b**) and (**c**) show the average error and average distance as functions of the number of permutations (*n*) and the length threshold of a CBB ($L_{CBB}$), respectively. The results are obtained from an experiment with 1 million CBBs computed for (Id=0,...,999999). In Fig. (**c**), the results are depicted as a linear regression line. Moreover, to check for similarities among CBBs, we used LSH with parameters ($k=4, r=200, b=20$). (**b**) shows how the average error reduces, and the average distance increases with an increasing number of permutations. (**c**) illustrates how the error decreases and relative distances increase with the length of CBBs. In (**d**), we illustrate the effects of segmentation on the average error and average distance. We encoded the first 1 million rows ($\approx 600$ megabytes) of the data set A321_valid.csv and set the parameters such that the resulting Info-DNA sequences after segmentation are of length $L_{Info}=170$. We encoded the unique attribute Id and the non-unique attribute icao24 as CBBs. The CBBs are all of length $L_{CBB}=80$ (padded by $Pad(cbb, L_{CBB})$, see "Methods"). We generated a total of $\approx 15.3$ million oligos (every oligo is of length $L_{Oligo}=250$) in the case with segmentation. We used 32 permutations for CBBs and segmentation, and used $c=10$, i.e., the number of base pairs to pad every Info-DNA segment with, in the segmentation case. Without segmentation, we generated 1 million oligos for encoding Id, each representing a complete data record, and 1044 oligos for encoding icao24. Thus, the sizes of these artificial oligos are not bounded to 250 base pairs anymore but range between 192 and 3018 (average $\approx 2185$). We used LSH with parameters ($k=6, r=200, b=20$) and set the number of permutations to $n=32$ for CBBs. Our results in (**d**) show a considerable improvement in the distances for the oligos. As expected, segmentation has almost no impact on CBBs. In fact, it is interesting to see that the quality of CBBs does not worsen though many more CBBs are required in case of segmentation. However, the error of the oligos with segmentation is slightly higher than the ones without segmentation. To combat this deficiency, we could increase the number of base pairs padded to the Info-DNA sequences (*c*) or the number of permutations (*n*), consequently raising the computation time.

### Content-based filter queries with CBBs

This section describes how CBBs enable content-based filter queries over a set of oligos $\mathbb {O}$ on DNA. Given a set of CBBs that qualify for a query, the basic idea is to use hybridization to extract the associated oligos from $\mathbb {O}$. Though hybridization can be performed in many different ways^22,24,29^, we refer to microarrays as the underlying hybridization technique without loss of generality. Using a CBB and a microarray, the search for the associated oligo follows the standard procedure. For example, for a DNA library $\mathbb {O} =\{(\texttt {\underline{ACGT}AGTA}), (\texttt {\underline{GTTA}GCCA}), (\texttt {\underline{GAAT}CGAT})\}$ where the underlined bases resemble the CBBs, we can fetch any oligo $o \in \mathbb {O}$ by printing its corresponding CBB (or the CBB’s complement because our DNA is double-stranded) onto a microarray. After printing, we pour the DNA library over the array to allow the oligos to hybridize to the printed CBBs. Finally, we wash the array and sequence the remaining hybridized (bonded) oligos. The washing procedure removes all oligos that failed to bind to the microarray. For example, printing (ACGT) onto a microarray allows fetching $(\texttt {ACGTAGTA}) \in \mathbb {O}$.

### Encoding relational tables on DNA

The essence of our approach is the generation of the codes for CBBs and Info-DNA. For CBBs, we introduce two encoding strategies for the two cases (i) encoding unique attributes, and (ii) encoding non-unique attributes. The first enables encoding a unique attribute, i.e., the values in the corresponding column are unique. The second allows an attribute to be encoded, even if a value in the column occurs multiple times in different records. All attributes (unique and non-unique) encoded in the CBB can occur in predicates of a filter query. Let us first assume that the length threshold $L_{CBB}$ is sufficiently large to store the entire coded information of an attribute.

As illustrated in Fig. 2, encoding a relational table produces oligos that comply with the five essential biochemical constraints C1–C5 presented in "Methods". These constraints are crucial to generating stable DNA for synthesizing, storing, and sequencing with minimal errors. CBBs must be unambiguous, i.e., encoding the same information always leads to the same CBB because CBBs resemble unique keys that identify target oligos. Furthermore, CBBs adhere to constraints C1 and C2. The encoding scheme of CBBs is designed such that even for two marginally different pieces of information, the corresponding CBBs are likely to be very different, i.e., their similarity is relatively low, and thus constraint C3 is fulfilled. The actual length *q* of a CBB is generally smaller than the length threshold $L_{CBB}$. In order to approach the required GC content of $50\%$ (see constraint C1), our method adds certain padding information consisting of $L_{CBB} - q$ base pairs to the CBB. After that, the method generates *n* permutations for each CBB and selects the one with minimal expected sequencing errors.

For the coding of Info-DNA, we utilize a fountain code^25^ that adheres to the constraints C1 and C2, and enables forward error correction. To further obey the remaining constraints C4 and C5, we must limit similarities, i.e., overlaps among Info-DNA. Since accessing the DNA is done by hybridizing the target oligos using CBBs, the similarity of the oligos must be low to reduce cross-hybridization noise. If space is still available in the oligo, we again use padding to fill up Info-DNA with base pairs to improve its stability. In the rare case that an oligo cannot store the code of a record, our encoding method generates multiple oligos of length $L_{Oligo}$ for the record using segmentation (see "Methods"). For each of these smaller oligos, specific padding is added to improve its GC content to 50% if necessary. Finally, from a set of permutations, the one with the lowest expected error is selected with respect to the constraints. All these steps are reversible via meta-information, and thus the original record can be reconstructed. In summary, segmentation and permutations improve synthesizing and sequencing^30^.

We generated $\approx 15.3$ million oligos with segmentation for the experiment in Fig. 2d. The pairwise overlap computation of these DNA sequences induces an enormous overhead, and thus finding the mutual similarity of every two oligos is not practical. Instead, we use locality-sensitive hashing (LSH) to approximate the Jaccard distance^26–28,31^. The parameters for LSH (*k*, *r*, *b*) are further explained in the supplementary material. All experiments were conducted on a server computer with 1 TB RAM and 256 logical processing units (clock speed $1.5-2.25$ GHz).

Figure 2a illustrates the encoding algorithm. The attributes Id and Velocity are encoded into DNA. Id is the primary key, but Velocity allows duplicate values. This enables answering ad-hoc filter queries on the encoded attributes, and the method is detailed in the following section. Figure 2b shows how the overall error declines with an increasing number of permutations, and the average mutual distance grows for 1 million generated CBBs. In Fig. 2c, we average the error and distances for all permutations $n \in \{0, 2, 4, 8, 16, 32, 64\}$ by varying the length threshold $L_{CBB}$ for CBBs. Note that the actual length of a CBBs could vary for small $L_{CBB}$ in particular. For example, for $L_{CBB} = 0$, there is no padding because the size of a CBB is obviously larger than $L_{CBB}$. Furthermore, the errors are smaller, and the average distances becomes higher with an increasing $L_{CBB}$ (see "Methods"). There are two reasons for this. First, it is more likely to improve the GC content for larger $L_{CBB}$. Second, a larger $L_{CBB}$ also leads to more diverse sequences when permutations are generated.

We studied the distance and error rates of the following DNA converters (see "Methods"): NaiveQuattro, RotatingQuattro, RotatingTre, and Bin. Noticeably, our introduced DNA converter RotatingQuattro yields the highest average distances of the generated CBBs even for small $L_{CBB}$. Despite that, RotatingTre generates CBBs with the lowest error at the expense of higher similarities compared to RotatingQuattro. Hence, we recommend using RotatingTre to encode smaller data sets and RotatingQuattro for larger data sets to keep the similarities low and reduce contention for random access. The DNA converters Bin and NaiveQuattro perform worst in maximizing distances. Finally, Fig. 2d indicates that applying segmentation could reduce the overall similarity among oligos. The used error function is further detailed in the supplementary material.

### Answering ad-hoc filter queries on DNA

**Figure 3:**
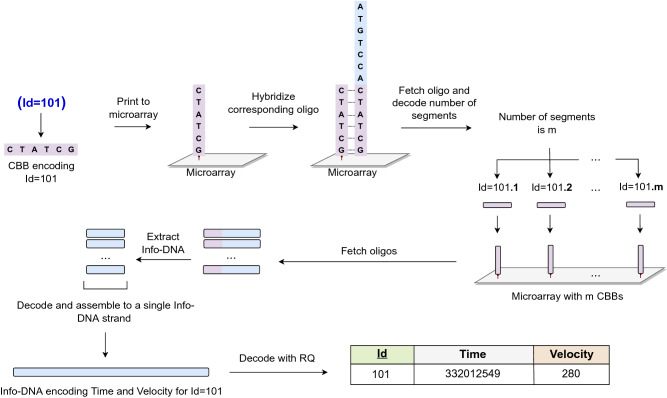
The figure depicts an example of how to answer the filter query (Id=101) on the unique attribute Id in Fig. 2a that is encoded with segmentation. First, the predicate given in the query (Id=101) is printed on a microarray, and the matching oligo is fetched. This strand contains the number of segments *m* that encodes the desired record. After decoding it, the remaining segments are retrieved by fetching the CBBs encoding (Id=101.i) for $1 \le i \le m$. After that, we decode each DNA sequence and assemble all the segments (without the CBBs) into a single Info-DNA sequence. The assembled Info-DNA strand encodes the information (Time=332012549, Velocity=280) that is decoded with RQ, and the desired record is returned. Note that every segment is decoded such that the used permutation is reversed and the padding removed.

Our encoding scheme allows answering filter queries that match an attribute to a given value. Given a table *R* of records with an attribute *A* and a value *v*, a filter query returns the records of *R* satisfying $A = v$. In order to run the query on DNA without reading all records of *R*, the attribute *A* must be encoded as CBB with our encoding method prior to querying. Furthermore, we distinguish answering queries on unique and non-unique attributes as detailed in "Methods". A filter query on a unique attribute requires at most two sequential read operations, regardless of how large the desired record is. To illustrate this, consider the following cases: (i) the unique attribute is encoded without segmentation, and (ii) the unique attribute is encoded with segmentation. In the first case, the desired record is fetched in one read operation by encoding the filtering condition as CBB, which is then used to read and decode the associated Info-DNA. For the second case, we first need to find the total number of oligos encoding the record. This information is stored in the first oligo segment as detailed in "Methods". This number is also sufficient to generate the CBBs for all the oligos encoding that record. Thus, after fetching the first oligo, we can fetch all the remaining oligos in parallel, yielding a total of two sequential read operations. Figure 3 provides an example for this procedure.

To answer the same query on a non-unique attribute A, let us first sketch the organization of the data. For each value *v* of attribute A in table *R*, we encode *v* as CBB of an oligo and keep the list of primary keys of the records with value *v* as its Info-DNA. As this list might be very long, segmentation has to be applied. In addition, there is an oligo for each record of the table, where Id serves as CBB and the record as Info-DNA. Similar to the procedure explained above, our method fetches the oligo (or multiple oligos in the case of segmentation) associated with the value *v* given in the filter predicate (see "Methods"). After decoding the DNA, we obtain the list of Id values that unambiguously identify the desired records. Finally, in the same way as for querying a unique attribute, our method fetches in parallel the records whose Id values are in the list. This is followed by decoding the DNA and returning the records. Hence, a query on a non-unique attribute requires at most four sequential reads. The first two are required to create the list of Id values, and the last two serve for retrieving the corresponding records for every Id in parallel.

## Discussion

Various methods are proposed to randomly access data objects on DNA^8,10,20,21,29,32–34^. Most of them utilize PCR to amplify target DNA strands for reading. However, these systems only allow random access by predefined DNA sequences, e.g., primers or barcodes that serve as addresses. Hence, it is possible to read the associated data object via an address, but there is no support for arbitrary ad-hoc filter queries on attributes. In addition, the primer or barcode sequences are stored on a different storage device (not DNA) to look up prior to querying on DNA. Our new method encodes structured data on DNA and enables accessing data objects based on their content. Our DNA is composed of two parts: (i) a content-based barcode (CBB), and (ii) an information-carrying part (Info-DNA). Furthermore, we introduce two encoding methods for answering ad-hoc filter queries on DNA. Our encoding scheme relies on utilizing CBBs that serve as addresses to enable random access by hybridization and facilitate content-based look-ups. These CBBs are computed in an ad-hoc fashion and do not require information to be stored *in-silico* on a traditional storage device. Our methods further allow answering ad-hoc queries that can return multiple data objects from DNA and are not limited to the key-value principle of primers or traditional barcodes.

For Info-DNA that is encoding information, e.g., a data record, we use a fountain code that enables variable forward error correction. CBBs and Info-DNA are encoded to avoid error-prone sequences such as homopolymers and other kinds of errors. Moreover, we propose segmentation of long oligos, a method that could further reduce the errors of DNA. This method allows encoding arbitrary digital data into DNA sequences of a predefined length, reducing the cost of synthesizing. In addition, we use segmentation of oligos to minimize DNA overlaps to reduce cross-hybridization noise. To efficiently compute DNA overlaps of many DNA sequences, we utilize locality-sensitive hashing, a method that approximates the Jaccard distance of DNA sequences with low computational overhead.

We show that our methods enable random access for several million content-based addresses and support various kinds of content-based filter queries. For a proof of concept, we generated $\approx 15.3$ million DNA sequences that fulfill constraints mentioned in "Methods" encoding a data set ($\approx 600$ megabytes in size) with one million records, each of them consisting of 56 attributes. Our encoding scheme enables filtering records on DNA based on matching a column’s value. For example, we could fetch every record where the attribute Velocity equals 512. We sent our generated DNA to Twist Bioscience (https://www.twistbioscience.com/), which confirmed our DNA was ready for production.

Our introduced DNA converter RotatingQuattro best minimizes the mutual similarities among CBBs compared to other proposed converters. By permuting CBBs and Info-DNA sequences, we avoid generating error-prone DNA and significantly reduce the overall error. Our encoding scheme allows setting and tweaking multiple parameters such as the used error function (see "supplementary material"), the threshold length of CBBs ($L_{CBB}$), the target length of every oligo ($L_{Oligo}$), the number of permutations used to encode Info-DNA (*n*), the parameters for the LSH method, which ultimately affect the computation time and DNA quality.

Even though our approach is the first to support content-based search in large databases on DNA, there is potential for improvements. First, it requires the filter attributes to be known prior to encoding. Second, the filter queries only fetch records by an exact match of an attribute’s value. For example, we can find records where the predicate is $(\texttt {age = 30})$ but are not able to support range predicates like $\texttt {age} \ge \texttt {30}$. In our future work, we will work on mitigating these deficiencies. In particular, we currently study how to extend our approach to supporting range predicates. We also plan to conduct a proof of concept on real DNA, exploring its actual feasibility and scale.

## Methods

### Biochemical constraints on DNA

Today’s sequencing technologies allow near-perfect DNA synthesis for thousands of DNA fragments in parallel. Sequencing machines can reduce errors by reading a DNA strand from both ends and allowing multiple reads of the same sequence. However, specific sequences are more error-prone than others and could result in unrecoverable errors. Even a small error can lead to a significant decrease in product quality and has to be considered. In order to minimize errors, an encoding scheme must obey the following five constraints for DNA:

1. GC content (number of G’s and C’s) should be around 50%.
2. Consecutive repeats of the same nucleotide (*homopolymers*) should be minimized.
3. Similarities among barcodes like CBBs should be minimized.
4. Similarities among Info-DNA sequences should be minimized.
5. Similarities among the final DNA strands (oligos) should be minimized.

Considering C1, DNA sequences with a too low or too high GC content are known to be less stable and thus have to be avoided^35,36^. C2 is necessary because long consecutive repeats of the same nucleotide destabilize DNA strands, and sequencing machines fail to read them correctly^11^. C3, C4, and C5 minimize cross-hybridization noise among the entire DNA library. Too similar DNA strands would compete while hybridization^37,38^ and might alter the DNA, or at best produce false positives^39,40^.

### Padding DNA (GC content optimization)

The GC content seriously impacts the sequencing error. To keep the error small, the GC content of a sequence should be close to $50\%$. Unfortunately, this is not fulfilled for most of the codes. In order to improve the GC content of a sequence, e.g., CBB or Info-DNA, we pad additional base pairs to the sequence as outlined in the following. Let *seq* be a DNA sequence with length smaller than a threshold *L* ($|seq| < L$). We then pad base pairs to *seq* to obtain a new sequence $\widehat{seq}$ of length *L* and a GC content closer to $50\%$ compared to the one of *seq*. For the padding, eleven pre-computed DNA sequences $p_0,\ldots ,p_{10}$ are used, of which the GC content range (in 10% steps) between 0% and 100%. One of the eleven sequences is selected for padding such that the expected GC content of $\widehat{seq}$ is closest to $50\%$. In order to support very small sequences, there needs to be at least $L - 1$ base pairs in each of the eleven pre-computed sequences used for padding. Note that we only apply padding to *seq* if $|seq| < L$.

The index *i* of the pre-computed DNA padding sequence $p_i$ is computed in the following way:

1 $$\begin{aligned} i = \max \left\{ {0,\left\lfloor {10 \cdot \frac{{\frac{L}{2} - |seq{|_{\{ G,C\} }}}}{{L - |seq|}}} \right\rfloor } \right\} \end{aligned}$$

For a sequence *s* and $\texttt {B} \subseteq \{\texttt {A,C,T,G}\}$, $|s|_{B}$ returns the absolute number of $b \in \texttt {B}$ in *s*.

In our setting, padding is applied to CBBs and Info-DNA. For CBBs, we set the threshold $L_{CBB}$ for CBBs sufficiently large such that $|CBB| \le L_{CBB}$ holds in all cases, where *CBB* is the CBB before padding and permuting. For Info-DNA, the threshold is $L_{Info}$ and $|\text {Info-DNA}| \ge L_{Info}$ might be possible. This case is discussed in full detail in section "Segmentation". To distinguish these two cases, we use *marker base* (C), if Info-DNA fits into an oligo, and *marker base* (A) otherwise.

In order to decode the resulting sequence $\widehat{seq}$ back to *seq*, we append a unique delimiter sequence to *seq* before appending the base pairs from the pre-computed sequence $p_i$. This delimiter sequence is not found in any of the pre-computed sequences. Therefore, the delimiter can be unambiguously located in $\widehat{seq}$ that allows *seq* to be reconstructed.

Though $\widehat{seq}$ has an improved GC content, its distribution is non-uniform. In particular, the prefix of $\widehat{seq}$ is equal to *seq*, and thus the GC content in this part did not improve. To obtain a more uniform distribution of the GC content, we use an appropriate permutation of $\widehat{seq}$ (see next section).

Let us consider an example to illustrate our approach for $L=12$. For $seq=(\texttt {GTGTTA})$, the length is 6 and GC content is $\approx 33.3$%. Let us append the delimiter (AC) and the marker base C to *seq* that result in a new sequence $(\texttt {CGTGTTAAC})$ with GC content 44.4%. Then we append 3 base pairs from the pre-computed sequence $p_6$ that has a GC content of at least 60% according to 1. Let $p_6=(\texttt {CAGTCGGCGAGA})$ be the pre-computed padding sequence, the resulting sequence $\widehat{seq}=(\texttt {CGTGTTAACCAG})$ has a GC content of 50% and length $L=12$.

We name the function described above *Pad* and use $\widehat{seq} = Pad(seq, L)$ to express that *seq* is transformed into $\widehat{seq}$ under the constraint that $|\widehat{seq}| = L$. Furthermore, the inverse function $Pad^{-1}$ expresses the decoding, i.e., $seq = Pad^{-1}(\widehat{seq})$.

### DNA permutation (avoidance of long homopolymers)

Long homopolymers in DNA sequences cause high errors and must be avoided. Moreover, it is important to obtain sequences with a GC content that is as uniform as possible. In order to achieve these goals, we create *n* permutations $\pi _{k}$ of the original sequence *seq*, $1 \le k \le n$, and select the one whose distribution is closest to uniform. Formally, a *permutation* of a sequence *seq* is expressed as a list of index pairs (*i*, *j*) that are swapped in *seq*, i.e., the base pair at position *i* of *seq* is swapped with the one at position *j*. In our method, we used the classical Fisher-Yates shuffling algorithm with $|seq|-1$ swaps^41^. Then, the sequence of swaps is given by $(i, {rand}_i)$ where ${rand}_i \in \{0,\ldots ,i\}$ and $i=n-1,\dots ,1$ is randomly selected. This method guarantees the randomness of the resulting sequence (see "supplementary material").

For example, applying the permutation [(3, 2), (2, 2), (1, 0)] to the sequence (ACTC) results in the permuted sequence (CACT). To reverse a permutation, we again generate the corresponding list of index pairs and swap every index pair in reverse order starting from the last pair and ending with the first pair.

Furthermore, the index pairs are computed from a random numbers generator that is initialized with the following *seed*:

2 $$\begin{aligned} seed = |seq{|_{\{ A\} }} \cdot |seq{|_{\{ C\} }} \cdot |seq{|_{\{ T\} }} \cdot |seq{|_{\{ G\} }} \end{aligned}$$

It is important to note that *seed* is independent of the permutation of the sequence but likely to differ for different sequences. This gives us two advantages. First, it is possible to map a permuted sequence back to the original one by simply computing the inverse swaps. Second, two similar sequences result in permutations that are not similar anymore with high probability. Because we need a total of *n* different permutations $\pi _k$ for a sequence *seq*, we also require different seeds to initialize the random generator. Hence, our method uses for the $k-th$ permutation $\pi _k$ the seed $seed_k = seed+k$, $0 \le k < n$, for the initialization of the random generator. In order to guarantee the computation of the original sequence from the $k-th$ permutation, we encode the offset *k* as a prefix sequence of the permutation. This is more detailed in our method called DNA Packing found in the "supplementary material". For decoding, we first compute the offset *k* from the prefix and then initialize the random generator using $seed + k$. This allows for recovering the original sequence.

As shown in Supplementary Fig. 1, CBBs encoded with our setting of seeds results in a significantly higher average mutual distance. Moreover, the different seeds of the permutations yield drastically different permutations even for slight variations in the number of A’s, T’s, C’s, and G’s of the sequences.

Let us consider a simple example to generate $n=3$ permutations of $seq=(\texttt {AGCTG})$. First, we compute $seed = 4$. Then, we initialize 3 RNGs with 4, 5, and 6 as seed, respectively. Let the index pairs generated by the first RNG be [(4, 2), (3, 3), (2, 0), (1, 1)] yielding $(\texttt {GGATC})$ as its permutation, by the second RNG [(4, 2), (3, 0), (2, 2), (1, 1)] yielding $(\texttt {TGGAC})$, and by the third RNG [(4, 1), (3, 1), (2, 2), (1, 0)] yielding $(\texttt {TACGG})$.

In order to select the most promising permutation, we rank the permutations based on an error function (see supplementary material) that is applied to each of them. The one with the lowest error is then selected for coding the DNA sequence.

### Encoding content-based barcodes (CBBs)

For an object of a table, a CBB is derived from the corresponding row and column. For example, the CBB encoding Id=100 would correspond to the value 100 of column Id, where Id is the primary key. A CBB would encode the value and its attribute’s name. Our encoding algorithm consists of the following 5 steps.

**Figure 4:**
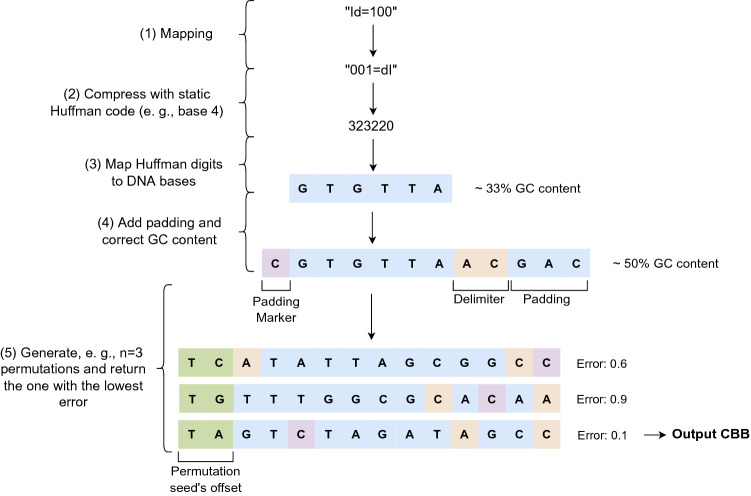
Encoding information as a CBB.

1. **Mapping**. The first step is to merge (and reverse) the desired value and its attribute’s name into a single string. As shown in Fig. 4, for a value 100 in the column Id, we create the string “001=dI”. The reversal of the string is performed because the DNA converters (step (3)) are sensitive to changes in the first characters and, thus, produce different DNA even if the input is similar.
2. **Compression**. The string obtained from the mapping step is then compressed byte by byte with a static Huffman code to produce a string of Huffman symbols. Since CBBs are unambiguous, the same attribute value must lead to the same CBB, and the used frequencies are fixed for all CBBs. Our frequencies are available in the supplementary material.
3. **DNA Conversion**. The resulting Huffman symbols are then mapped to DNA bases. There are many ways to convert these symbols to DNA bases that depend on the Huffman code’s base. We discuss the examined DNA converters RotatingTre, RotatingQuattro, Bin, and NaiveQuattro in the supplementary material. Figure 4 illustrates our converter RotatingQuattro that requires compressing “001=Id” with a Huffman code of base 4.
4. **DNA Padding (**GC ** Content Optimization)**. In this step, the obtained sequence *seq* from step (3) is padded using our function *Pad*(*seq*, *L*). This is illustrated for $L = 12$ in Fig. 4. Note that in this example, $L_{CBB} = 14$ due to the permutation’s seed offset that is added (see next step).
5. **Permutation (Avoidance of Long Homopolymers)**. After the padding step, the obtained sequence $\widehat{seq}$ has a GC content of $\approx 50$%, but could still suffer from long homopolymers and a non-uniform distribution of G’s and C’s. To further fulfill constraint C2, we permute the sequence as detailed above.

Note that constraints C3-C5 are not checked for CBBs to make them unambiguous. In other words, encoding the same information always leads to the same CBB sequence. For any two values *v* and *w* of the same column that are encoded as $CBB_{v}$ and $CBB_{w}$, respectively, the following equation holds:

3 $$\begin{aligned} v \ne w \Leftrightarrow CBB_{v} \ne CBB_{w} \end{aligned}$$

Even when values occurring in different attributes are equal, the computed CBBs are different because the names of the attributes contribute to the code. However, if values are equal in one column, the corresponding CBBs would be identical and violate constraints C3 and C5. To overcome this deficiency, we introduce two different methods to encode values of (i) a unique column, and (ii) a non-unique column in the following sections.

### Encoding info-DNA with redundancy

#### Background

RaptorQ code (RQ) is a fountain code^25^ that encodes a *k*-symbol message into a potentially infinite number of encoded symbols called packets (a packet can contain multiple encoded symbols). It allows efficient forward error correction and is typically used to send messages over lossy communication channels. Because of unreliable networks, some packets of a sender might never reach the receiver. In order to achieve successful communication, RQ introduces error correction such that the sender could send a potentially infinite sequence of packets until it captures a stop signal from the receiver stating that the entire message was successfully decoded.

The main advantage of RQ is that the receiver can decode the message with high probability if any $k+\varepsilon$ arbitrary packets have arrived. The term $\varepsilon$ is called the *overhead* or *redundancy* and is typically a very small number. For a source message with *k* source symbols $(s_1, s_2,...,s_k)$, RQ attempts to send the first *k* packets $(p_1,p_1,...,p_k)$ that are equal to the source symbols, i.e., $p_i=s_i, 1 \le i \le k$. If the first *k* packets are sent, and the sender did not obtain a stop signal, the sender will continue sending *repair* packets that are computed from a range of source symbols. The *repair* packets compensate for any of the lost packets. Even if the first *k* packets of RQ are lost, and only the following repair packets are received, it is still possible to decode the message with the same high probability. This is called a *non-systematic* code. For example, setting $\varepsilon = 2$, the probability of successfully restoring the *k*-symbol source message from $k + 2$ packets is greater than 99.9999%.

**Figure 5:**
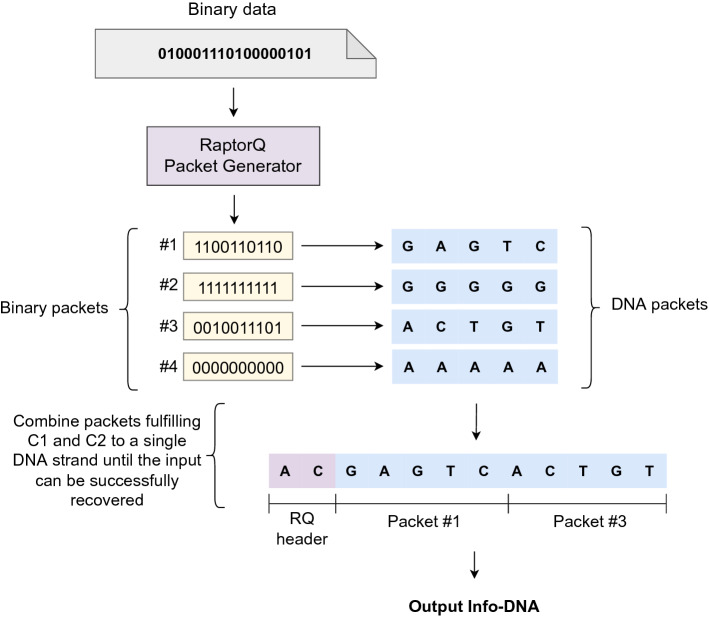
Encoding information with RaptorQ code.

#### Packet generation

RQ takes a binary message as input and outputs a stream of binary packets. To convert the binary packets to DNA, we map every two consecutive bits to a respective DNA base. This conversion is exemplified in Fig. 5, where the binary packet (11 00 11 01 10) results in (GAGTC) by using $\texttt {00}\mapsto \texttt {A}, \texttt {01}\mapsto \texttt {T}, \texttt {10}\mapsto \texttt {C},$ and $\texttt {11}\mapsto \texttt {G}$. If the original source message contains long runs of 0’s or 1’s, it causes the problem of homopolymers again. For example, the source message (11 11 11 11 11) is mapped to (GGGGG). In general, homopolymers occur less frequently in repair packets as they are computed from various source symbols^42^. Therefore, we use repair packets only in our approach. Moreover, by increasing the redundancy parameter $\varepsilon$, we can generate more packets than actually required to decode the message in the case of DNA errors.

#### Filtering and combining packets

As depicted in Fig. 5, a binary source message is fed into the RQ’s packet generator that outputs binary repair packets. Then, each binary packet is mapped to DNA, as discussed above. Since RQ can create many packets with little computational overhead, we only send the stop signal once we capture a sufficient number of repair packets that (i) fulfill constraints C1 and C2, (ii) allow the source message to be recovered, and (iii) contain sufficient redundancy information to compensate for synthesizing/sequencing errors. In Fig. 5, RQ generates 4 packets, of which packet $\#1$ and packet $\#3$ fulfill constraints C1 and C2, and allow the source message to be recovered. The following section details how constraints C4 and C5 are addressed.

In order to decode DNA packets, we insert an *RQ header* that contains the meta-information required for the RQ decoder. The explicit structure of DNA packets is shown in supplementary Fig. S2, and the RQ header is further explained in the supplementary material.

### Segmentation

We consider oligos with a fixed size $L_{Oligo}$ of base pairs. Each oligo comprises two parts, one for storing CBB and the other for Info-DNA. A CBB of length ($L_{CBB}$) contains only a value of an attribute, while Info-DNA (of length $L_{Info}$) encodes an entire record or a list of identifiers (see next sections). Because attribute values occupy a few bytes, it is justified to assume that a CBB only requires a few base pairs (including those for padding). In our experiments, the setting $L_{CBB} = 60$ was sufficient to store all the attribute values in a CBB and still allow padding. However, the size of Info-DNA can easily exceed $L_{Oligo} - L_{CBB}$, which is the remaining size for Info-DNA. Then, it is required to partition the information into segments, each of which is kept in its own oligo. In the following, we present our method for segmenting Info-DNA among *m* oligos, $m>1$.

Supplementary Fig. S3 provides an example of the method. As shown, we consider the second record of the table in Fig. 2 where (Id=101) and (Time=332012549, Velocity=280) is encoded as CBB and Info-DNA, respectively. For the sake of illustration, this oligo is set artificially short. For a given long Info-DNA sequence *seq*, we use $seq_i$ to denote the part of *seq* assigned to the *i*-th oligo.

In order to compute the segmentation of *seq*, let us recall that our coding method uses free space for padding to improve the GC content and additional space for storing the index of the selected best permutation. Let *c* and *r* denote the number of base pairs reserved for padding and permutation, respectively. Then, the number *m* of segments is given by:

4 $$\begin{aligned} m = \Bigg \lceil \frac{|seq|}{L_{Info} - c - r}\Bigg \rceil \end{aligned}$$

The first oligo stores *m* as the only information in Info-DNA. Then, there are *m* subsequent oligos storing $seq_i$ (plus the extra base pairs for padding and permutation) in its Info-DNA. The subsequence $seq_i$ of *seq* starts at index $(i-1) \cdot \Big \lceil \frac{|seq|}{m} \Big \rceil$ for $i = 1,\ldots , |seq| \mod m$ and contains $L_{Info} - c - r$ base pairs of *seq*. Note that the resulting Info-DNA of $seq_i$ contains additional *c* base pairs for padding and *r* base pairs for the permutation offset, resulting in a total of $L_{Info}$ base pairs. For example, let *seq* be an Info-DNA sequence of length 20 and $m = 5$. This setting results in 5 subsequences $seq_1, \ldots , seq_5$ where $seq_2$ contains the base pairs 4, 5, 6, 7 of *seq* and $seq_4$ the base pairs 12, 13, 14, 15.

Finally, we discuss the generation of the CBBs for the oligos to enable accessing their associated segments. Let us consider the example in Supplementary Fig. S3. The first oligo consists of the original CBB encoding (Id=101). This CBB serves as a template for the remaining *m* CBBs. For segment $seq_i$, we encode (Id=101 **.i** ) as its CBB, $1 \le i \le m$. The encoding algorithm of CBBs ensures that the suffixes ${\textbf {.i}}$ generate substantially different CBBs, even though the inputs are quite similar.

To retrieve the desired information from such a segmented list of oligos, the original CBB is used to fetch the first oligo whose Info-DNA contains *m*, the number of oligo segments. After successful decoding, we simultaneously fetch all the remaining *m* oligos using the derived CBBs, e.g., (Id=101.1), ..., (Id=101.m), reverse the permutation, remove the padding, concatenate them into one sequence *seq*, and finally decode *seq* with RQ. Note that the CBB encoding (Id=101.1) corresponds to the first segment, the CBB encoding (Id=101.2) to the second segment, and the CBB encoding (Id=101.m) to the last segment.

Let us finally discuss all the steps above in an example illustrated in Supplementary Fig. S3. By setting $L_{Info}=14, r = 1, c = 5, n = 3$, we generate a total of $m = \Big \lceil \frac{16}{14 - 5 - 1}\Big \rceil = 2$ additional oligo segments. After partitioning the input sequence into subsequences, we further pad each subsequence. The padding delimiter (AC) marks the first position where the padding begins. Padding does not fully exploit the entire free space but leaves some space available for the seed offset of the permutation ($r=1$ base in green) required to reverse the permutation. After the permutation step, we create the CBBs for the oligos. Note that every resulting Info-DNA segment is of length 14. The first Info-DNA segment that encodes *m* is marked with the CBB encoding (Id=101), the second Info-DNA segment is marked with the CBB encoding (Id=101.1), and the last Info-DNA segment is marked with the CBB encoding (Id=101.m).

### Encoding unique attributes

This section presents the method for encoding and retrieving records given a value of a unique attribute. For example, consider the filter query (Id=101) where Id represents the primary key in Fig. 2. The result consists of the record (Id=100, Time=1015430211, Velocity=512). Because Id is unique, no other record qualifies.

First, we detail how to encode a record to support filter queries on unique attributes. The basic idea is to encode the values of a unique attribute as CBB and the values of the other attributes of the record as Info-DNA. For example, the oligo of the record (Id=101, Time=1015430211, Velocity=512) consists of a CBB encoding the value (Id=101), and an Info-DNA encoding (Time=1015430211, Velocity=512). In more detail, there are multiple steps for encoding Info-DNA. First, the method Data Packing explained in the Supplementary Material transforms (Time=1015430211, Velocity=512) to a binary representation. After that, the binary sequence is compressed with GZIP^43^ and fed into RQ. Figure 2 shows how the first 4 records of the attribute Id of the table in Fig. 2 are encoded. Furthermore, segmentation is applied if Info-DNA is too long or one of the constraints C1, C2, C4, and C5 are violated. The resulting oligos contain an Info-DNA no longer than $L_{Info}$. Finally, an additional permutation step introduces more randomness in the sequences and fewer homopolymers.

This encoding scheme supports answering an arbitrary filter query with predicate (A=v) where the attribute A is unique and v is a value in A. In order to retrieve the record, we encode the condition as a CBB (assuming no segmentation), print it onto a microarray, and fetch the record that hybridizes to that CBB. If the query has no match, the microarray will not yield any hybridized sequence because there is not much overlap among oligos due to constraints C3–C5.

### Encoding non-unique attributes

This section presents the encoding and retrieving of records for a non-unique attribute. This is a prerequisite for supporting filter queries with predicates (A=v), where A is a non-unique attribute of a relational table *R*, and consequently, multiple records can qualify.

Encoding a non-unique attribute A in the same way as a unique attribute would lead to identical CBBs for different records. Thus, constraints C3 and C5 would be violated. Instead, we proceed as follows. First, we compute all the different values of attribute A. Let $a_1,\dots , a_d$ be the distinct values. For every $a_i$, we create a list $L_i$ with r.Id of records *r* that fulfill $r.A=a_i$. Recall that Id is the table’s primary key and thus a unique attribute. Then, we create an oligo for every $a_i$, $1 \le i \le d$, where $a_i$ is encoded as CBB. These CBBs are guaranteed to be different because of Eq. (3). Each computed list $L_{i}$ is compressed into a binary string by Delta+Range (see Supplementary Material) followed by GZIP. Finally, the compressed string is encoded as Info-DNA. We use segmentation if the resulting Info-DNA is too long or constraints C3-C5 are violated. For example, we depict the encoding of the first four records of the non-unique attribute Velocity in Fig. 2. There are two oligos: the first contains a CBB for (Velocity=512) and an Info-DNA encoding the list [100, 103]. The second oligo contains a CBB encoding (Velocity=280) and an Info-DNA encoding the list [101, 102].

To answer a filter query (A=v), our algorithm encodes the condition (A=v) as a CBB, prints it onto a microarray, and fetches the matching oligos. Then, RQ decodes the Info-DNA, and the obtained binary string is decompressed via GZIP and Delta+Range. The result contains the list of Id values that unambiguously denote which records match the given condition. Finally, every Id value of the list is encoded as a CBB that serves for reading the associated oligo from the microarray. A last decoding step of the oligo returns the desired record. Note that these final processing steps for Id values are performed in parallel. This is crucial for keeping the cost of our approach low.

## Supplementary Information

Supplementary Information.

## Data Availability

We used the data set from the OpenSky Network (https://opensky-network.org/) that contains tracking data of aircraft flying around the world. The data set contains 56 attributes such as Velocity, Time, and Heading. We inserted a new column named Id that assigns a unique number starting from 0 to every record. Hence, Id serves as the table’s primary key, i.e., the attribute is unique. The compressed data set named “A321_valid.csv.xz” can be downloaded from: (https://opensky-network.org/datasets/publication-data/climbing-aircraft-dataset/trajs/).

## Code availability

Our code is available at: https://github.com/alexelshaikh/dna-system.
