# Supplementary material for: Content-based filter queries on DNA data storage systems
Source: Sci Rep. 2023 Apr 29;13:7053. doi: 10.1038/s41598-023-34160-5 (PMC10148835; doi:10.1038/s41598-023-34160-5)
Supplement: Supplementary file 1 — Supplementary Information. [file 41598_2023_34160_MOESM1_ESM.pdf]

Supplementary Material for
Content-Based Filter Queries on DNA Data Storage Systems

Alex El-Shaikh[1,*], and Bernhard Seeger[2,*]
[1]elshaika@mathematik.uni-marburg.de
[2]seeger@mathematik.uni-marburg.de

[*]Department of Mathematics and Computer Science
University of Marburg, Germany

Contents

1 Notation **2**

2 Huffman Code's Frequencies **2**

3 DNA Converters **2**
- 3.1 NaiveQuattro . . . 3
- 3.2 RotatingQuattro . . . 3
- 3.3 RotatingTre . . . 4
- 3.4 Bin . . . 6

4 RaptorQ Code (RQ) Header Structure **6**

5 Locality-sensitive Hashing (LSH) **7**

6 Error Functions **8**

7 Fisher-Yates Permutations **9**

8 Data Packing **9**

9 DNA Packing **10**

10 Delta+Range Compression **10**

11 Supplementary Figures **11**

12 Supplementary Tables **13**

1 Notation

A DNA sequence is represented as a vector $seq = (b_0, b_1, \dots, b_{n-1}) \in \{\texttt{A,C,T,G}\}^n$ where the length of seq is denoted as:

$$|seq| = |(b_0, b_1, ..., b_{n-1})| = n \tag{1}$$

We write $(b_0 b_1 ... b_{n-1})$ instead of $(b_0, b_1, \dots, b_{n-1})$ for simplicity. For example, $|(\texttt{ACGT})| = 4$. To access a specific base at index i in seq, we calculate:

$$seq[i] = \begin{cases} b_i, & 0 \leq i < |seq| \\ b_{(i+|seq|) \ mod \ |seq|}, & -|seq| \leq i < 0 \end{cases} \tag{2}$$

By setting $seq = (\texttt{GATCT})$ we calculate $seq[0] = \texttt{G}$, $seq[-1] = \texttt{T}$, $seq[-2] = \texttt{C}$, and accessing $seq[5] = \_$ denotes that the index 5 is not defined in seq. To count the number of $b \in B \subseteq \{\texttt{A,C,T,G}\}$ in seq, we write:

$$|seq|_B = \big|\{i \mid seq[i] \in B \land 0 \leq i < |seq|\}\big| \tag{3}$$

For example, $|(\texttt{GATCT})|_{\{\texttt{G,C}\}} = 2$, $|(\texttt{GATCT})|_{\{\texttt{A}\}} = 1$, and $|(\texttt{GATCT})|_{\{\texttt{T}\}} = 2$. To compute the `GC` content (between 0% and 100%) of a sequence seq, we use the shortcut $\texttt{GC}(seq)$:

$$\texttt{GC}(seq) = \frac{|seq|_{\{\texttt{G,C}\}}}{|seq|} \tag{4}$$

For example, $\texttt{GC(GATCT)} = 40\%$ and $\texttt{GC(TTATA)} = 0\%$.

2 Huffman Code's Frequencies

Our DNA converters rely on compression of the input with Huffman code before mapping it to DNA. To make these DNA converters unambiguous, i.e., the same input is mapped to the same DNA every time, we use static frequencies to generate the same Huffman code. The code compresses each byte (ASCII) of a given string of bytes. Furthermore, the used frequencies favor the letter frequencies of written English. Note that any other frequency assignment would work but might yield a smaller compression ratio. For example, in our case, the letter e is assigned a higher frequency than the letter z. The full list of frequencies used can be downloaded from (`https://github.com/alexelshaikh/dna-system/blob/master/freqs.txt`). The file contains 256 lines, each mapping a byte to a frequency number. For example, `101=12702` assigns the frequency 12702 to the byte 101 ($\widehat{=}$ letter e).

3 DNA Converters

This section details the methods used to map digital bits to DNA by encoding content-based barcodes. To convert a stream of bytes (e.g., a string) to DNA, we implement 4 different methods: `NaiveQuattro`, `RotatingQuattro`, `RotatingTre`, and `Bin`, which we will explain in the following sections. All DNA converters take a string of characters (bytes) as input and output a DNA sequence.

3.1 NaiveQuattro

First, the input string is compressed with the static Huffman code of base four. The resulting Huffman code is a stream of Huffman digits. Each Huffman digit is then mapped to a DNA base as follows:

$$0 \leftrightarrow \texttt{A}$$
$$1 \leftrightarrow \texttt{T}$$
$$2 \leftrightarrow \texttt{C}$$
$$3 \leftrightarrow \texttt{G}$$

To reverse the conversion, we map each DNA base back to the respective Huffman digit and decompress the resulting Huffman digits with the static Huffman code.

Example

The string "Alex" is compressed with the static Huffman code of base four to `3301231112201` and then mapped to the DNA sequence (`GGATCGTTTCCAT`). Decoding the DNA sequence is done analogously by mapping each base to the respective Huffman digit and decompressing the digits to the original string.

3.2 RotatingQuattro

Similar to `NaiveQuattro`, first, this method compresses the input with the static Huffman code of base four. After that, each obtained Huffman digit is mapped according to the rules in Table 1. The i-th mapping depends on the $(i-1)$-th and $(i-2)$-th mappings, if available. The column `#` denotes the rule's number, `[-1]` denotes the previously mapped base, `[-2]` denotes the base preceding `[-1]`, `Digit` denotes the current Huffman digit, and `Base` denotes the resulting DNA base.

Example

To convert the string "Alex" to DNA, we first compress it with the static Huffman code of base four. The obtained Huffman digits are `3301231112201`. Finally, each digit is mapped to a DNA sequence *seq* according to the mapping rules in Table 1 by following these steps (*seq* is initialized empty):

1. - $seq[-2] = \_, seq[-1] = \_, digit : 3$
   - Matching rule `#`4 $\Rightarrow seq =$ (`G`)
2. - $seq[-2] = \_, seq[-1] = \texttt{G}, digit : 3$
   - Matching rule `#`20 $\Rightarrow seq =$ (`GC`)
3. - $seq[-2] = \texttt{G}, seq[-1] = \texttt{C}, digit : 0$
   - Matching rule `#`73 $\Rightarrow seq =$ (`GCT`)
4. - $seq[-2] = \texttt{C}, seq[-1] = \texttt{T}, digit : 1$

- Matching rule #66 $\Rightarrow$ $seq = ($GCTC$)$

5. - $seq[-2] =$ T$, seq[-1] =$ C$, digit : 2$
   - Matching rule #43 $\Rightarrow$ $seq = ($GCTCT$)$
6. - $seq[-2] =$ C$, seq[-1] =$ T$, digit : 3$
   - Matching rule #68 $\Rightarrow$ $seq = ($GCTCTG$)$
7. - $seq[-2] =$ T$, seq[-1] =$ G$, digit : 1$
   - Matching rule #46 $\Rightarrow$ $seq = ($GCTCTGT$)$
8. - $seq[-2] =$ G$, seq[-1] =$ T$, digit : 1$
   - Matching rule #82 $\Rightarrow$ $seq = ($GCTCTGTA$)$
9. - $seq[-2] =$ T$, seq[-1] =$ A$, digit : 1$
   - Matching rule #38 $\Rightarrow$ $seq = ($GCTCTGTAT$)$
10. - $seq[-2] =$ A$, seq[-1] =$ T$, digit : 2$
    - Matching rule #35 $\Rightarrow$ $seq = ($GCTCTGTATA$)$
11. - $seq[-2] =$ T$, seq[-1] =$ A$, digit : 2$
    - Matching rule #39 $\Rightarrow$ $seq = ($GCTCTGTATAG$)$
12. - $seq[-2] =$ A$, seq[-1] =$ G$, digit : 0$
    - Matching rule #29 $\Rightarrow$ $seq = ($GCTCTGTATAGG$)$
13. - $seq[-2] =$ G$, seq[-1] =$ G$, digit : 1$
    - Matching rule #78 $\Rightarrow$ $seq = ($GCTCTGTATAGGA$)$

The resulting DNA sequence is $seq = ($GCTCTGTATAGGA$)$. To decode it back to the Huffman digits, we iterate over each base $seq[i]$ (from left to right) in seq where we match $seq[i]$ with `Base`, $seq[i-1]$ with `[-1]`, and $seq[i-2]$ with `[-2]` in Table 1. We return the corresponding `Digit` and collect them in a string of digits. Finally, these digits are fed into the Huffman code's decompressor, and the original string "Alex" is recovered.

3.3 RotatingTre

This method [1] compresses the input string with the static Huffman code of base three and maps the obtained digits according to the rules in Table 2. The i-th mapping depends on the $(i-1)$-th mapping, if available. The column `#` denotes the rule's number, `[-1]` denotes the previously mapped base, `Digit` denotes the current Huffman digit, and `Base` denotes the resulting DNA base. The obtained sequence is guaranteed to have no homopolymers of length two or greater.

Example

To encode the string "DNA" with `RotatingTre`, it is compressed with the static Huffman code of base three. Then, each of the Huffman digits `10212202021` is mapped to a DNA base. The following steps show how to map these digits to a DNA sequence *seq*:

1. - $seq[-1] = \_, digit : 1$
   - Matching rule #2 $\Rightarrow seq =$ (G)
2. - $seq[-1] =$ G$, digit : 0$
   - Matching rule #7 $\Rightarrow seq =$ (GT)
3. - $seq[-1] =$ T$, digit : 2$
   - Matching rule #12 $\Rightarrow seq =$ (GTG)
4. - $seq[-1] =$ G$, digit : 1$
   - Matching rule #8 $\Rightarrow seq =$ (GTGA)
5. - $seq[-1] =$ A$, digit : 2$
   - Matching rule #3 $\Rightarrow seq =$ (GTGAT)
6. - $seq[-1] =$ T$, digit : 2$
   - Matching rule #12 $\Rightarrow seq =$ (GTGATG)
7. - $seq[-1] =$ G$, digit : 0$
   - Matching rule #7 $\Rightarrow seq =$ (GTGATGT)
8. - $seq[-1] =$ T$, digit : 2$
   - Matching rule #12 $\Rightarrow seq =$ (GTGATGTG)
9. - $seq[-1] =$ G$, digit : 0$
   - Matching rule #7 $\Rightarrow seq =$ (GTGATGTGT)
10. - $seq[-1] =$ T$, digit : 2$
    - Matching rule #12 $\Rightarrow seq =$ (GTGATGTGTG)
11. - $seq[-1] =$ G$, digit : 1$
    - Matching rule #8 $\Rightarrow seq =$ (GTGATGTGTGA)

The resulting DNA sequence is $seq =$ (GTGATGTGTGA). Similar to `RotatingQuattro`, to decode it back to the Huffman digits, we iterate over each base $seq[i]$ (from left to right) in *seq* where we match $seq[i]$ with `Base`, and $seq[i-1]$ with `[-1]` in Table 1. We return the corresponding `Digit` and collect them in a string of digits. Finally, these digits are decompressed with the static Huffman code, and the original string "DNA" can be recovered.

3.4 Bin

As the name suggests, this method compresses the input with the static Huffman code of base two, generating a binary stream of Huffman digits. We map every two consecutive digits to a DNA base as follows:

$$00 \leftrightarrow \texttt{A}$$
$$01 \leftrightarrow \texttt{T}$$
$$10 \leftrightarrow \texttt{C}$$
$$11 \leftrightarrow \texttt{G}$$

If the resulting number of digits is odd, we will fail to map the last digit. Hence, we examine the following cases:

- Number of digits is even: Mark the resulting DNA sequence with the prefix base `A`.
- Number of digits is odd: Map every two consecutive digits according to the mapping rules above.
  - If the remaining digit is `0`, mark the resulting DNA sequence with the prefix base `G`. The last digit is not further mapped.
  - Otherwise, mark the resulting DNA sequence with the prefix base `C`. The last digit is not further mapped.

Example

To encode "PCR", we compress it with the static Huffman code of base two to `111011010101111101100`. Since the number of digits is odd, every two consecutive digits (excluding the last one) are mapped to seq = (`GCGTTTGGTC`). The last digit is `0`, thus, we insert the prefix base `G` yielding seq = (`GGCGTTTGGTC`). To decode the DNA sequence, we match the first base $seq[0]$. In this case, $seq[0]$ = `G` means the original sequence of digits contains an odd number of digits, of which the last digit is `0`. Therefore, we map each base (excluding the last one) to a digit according to the rules above and add a `0` to its end.

4 RaptorQ Code (RQ) Header Structure

RQ generates DNA packets for a byte input sequence *input* and combines the packets that adhere to the defined constraints to a single DNA sequence. Each packet contains a certain number of encoded symbols. We set the default number of encoded symbols per packet to:

$$\frac{|input|}{8} \quad (5)$$

where $|input|$ returns the number of bytes in *input*. The structure of a DNA sequence generated by RQ is illustrated in Figure 2. We refer to the first five bases as the outer header. It contains the number of bytes of the input and the desired number of encoded symbols $symbolSize$. The following bases resemble

the packets contained in this DNA sequence, i.e., Info-DNA. In this example, the strand contains exactly two packets. All packets begin with a *symbolSize identifying* base. If this *symbolSize identifying* base is `A`, then the number of encoded symbols in this packet is equal to *symbolSize* in the outer header as shown in packet #1. Otherwise, the next few bases represent the *symbolSize* of the packet as shown in packet #2. The following bases in purple encode the Forward Error Correction (FEC) payload id as defined in Request for Comments (RFC) 6330 (`https://datatracker.ietf.org/doc/html/rfc6330`). The following bases in green contain the encoded symbols. Note that we can read to the end of the block of encoded symbols because we previously decoded the *symbolSize*. Numbers such as $|input|$ and *symbolSize* are encoded to DNA by DNA packing that we explain in section 9.

5 Locality-sensitive Hashing (LSH)

According to constraints C3-C5 in the main manuscript, DNA overlaps should be minimized to reduce contention and enable random access. There are many similarity metrics to calculate the similarity of two DNA sequences, such as the Hamming distance, Edit distance, and Jaccard distance. Note that the distance is the complement of the similarity. For example, if two sequences are identical, the corresponding similarity would be 1 and the distance 0. Since computing DNA distances for many DNA sequences induces a significant overhead, we approximate distance calculations using locality-sensitive hashing (LSH). We implement LSH to approximate the Jaccard distance according to [2] where the parameter k sets the k-mer length, r the number of hash functions, and b the number of bands. For a given sequence seq, LSH can find similar sequences in a set of sequences $\mathcal{D}$ with high probability. First, we insert every sequence $seq_{\mathcal{D}} \in \mathcal{D}$ into the LSH table T_{LSH}. After that, we find similar sequences to seq as follows:

$$candidates(seq, T_{LSH}) \mapsto Q \subseteq \mathcal{D} \tag{6}$$

where the following conditions hold:

$$d(seq, seq_{\mathcal{D}}) \le t \quad\;\; \Rightarrow P[seq_{\mathcal{D}} \in Q] \ge p_1 \tag{7}$$

$$d(seq, seq_{\mathcal{D}}) \ge c \cdot t \Rightarrow P[seq_{\mathcal{D}} \in Q] \le p_2 \tag{8}$$

where t is the distance threshold, d the Jaccard distance function, P the probability function, and $c > 1$ an approximation factor. The probability of finding a sequence $seq_{\mathcal{D}} \in \mathcal{D}$ with $d(seq, seq_{\mathcal{D}}) \le t$ is at least p_1, and the probability of finding a sequence $seq_{\mathcal{D}} \in \mathcal{D}$ with $d(seq, seq_{\mathcal{D}}) \ge c \cdot t$ is at most p_2. Hence, p_1 is usually large and p_2 small. Furthermore, we define the minimum distance of a sequence seq to a set of sequences $\mathcal{D}$ using LSH as (assuming we inserted $\mathcal{D}$ into T_{LSH}):

$$\hat{d}(seq) = min\Big(\big\{d(seq, can) \mid can \in candidates(seq, T_{LSH})\big\} \cup \big\{1\big\}\Big) \tag{9}$$

The function above calculates the minimum distance of seq to every candidate match given by T_{LSH}. This function is used when we refer to calculating the distance of a sequence to a collection of sequences using LSH.

6 Error Functions

In general, an error function takes a DNA sequence as input and returns an error value between 0 and 1. Our error function is composed of multiple intermediate error values $e_1, \dots, e_k \in [0, 1]$. In order to achieve a higher impact on the total error, if one of the intermediate errors is high, the sum of the intermediate errors is normalized by the following function:

$$norm(e_1, \dots, e_k) = C_k \cdot \frac{1 + \frac{1}{\sqrt{e}}}{1 + \exp\left(\frac{1}{2} - 3 \cdot \sum_{i=1}^{k} e_i\right)} - \frac{1}{\sqrt{e}} \tag{10}$$

Note that e is the Euler's number and approximately equal to 2.71828. Furthermore exp denotes the exponential function with base e. The weighting constant C_k guarantees that the function $norm$ returns 1, given that every of the n input error values is equal to 1. Overall, $norm$ guarantees values being between 0 and 1.

Our error function $error(seq) = norm(e_1, e_2, e_3)$ comprises three intermediate error values e_1, e_2, e_3 to score the DNA sequences according to the given constraints. Value e_1 expresses the deviation of the `GC` content from 50%. Value e_2 accounts for the error regarding homopolymers, and value e_3 returns how the distribution of `G`'s and `C`'s in the sequence differs from a uniform distribution.

The following equation details the definition of e_1 for a sequence seq:

$$e_1(seq) = \begin{cases} 0, & |\mathtt{GC}(seq) - 0.5| \leq 0.05 \\ 0.4, & |\mathtt{GC}(seq) - 0.5| \leq 0.1 \\ 0.8, & |\mathtt{GC}(seq) - 0.5| \leq 0.2 \\ 1, & \text{otherwise} \end{cases} \tag{11}$$

Due to its design, intermediate error e_1 is increasing when the `GC` content of seq deviates further from 50%. If the `GC` content is lower than 30% or higher than 70%, e_1 already returns the highest error value 1.

For e_2, we assume that a homopolymer length up to $hp_{max} = 6$ is allowed in a sequence. Recall that a homopolymer is a subsequence sub_1 of seq consisting of only one symbol. For a subsequence sub_1 that is a homopolymer longer than hp_{max}, we ignore all homopolymers $sub_2 \subset sub_1$ and only consider sub_1. Let m be the number of homopolymers in seq of length larger than hp_{max} and let $h_1, \dots, h_m$ denote these homopolymers. Then e_2 is given as follows:

$$e_2 = norm\left(\frac{1}{m} \cdot \sum_{i=1}^{m} \left(1 - \frac{1}{1 + \exp\left(|h_i| - hp_{max}\right)}\right)\right) \tag{12}$$

Note that the resulting value is normalized using Equation 10 for $k = 1$.

The third intermediate error e_3 reflects how different the distribution of `C`'s and `G`'s is from a uniform distribution. The details are found in our implementation (in `SuperBasicRules.java`).

For Info-DNA (with segmentation), we choose the best among n permutations. For that, we consider the following score and choose the permutation with the largest score:

$$score(seq) = -c_1 \cdot error(seq) + c_2 \cdot \hat{d}(seq) \tag{13}$$

where *error* is the error function described above, and $\hat{d}$ is our LSH distance in Equation 9. The weighting factors $c_1 \geq 0, c_2 \geq 0$ can be adapted to favor either distance maximization or error minimization.

An implementation of the error functions is publicly available in our GitHub repository (`https://github.com/alexelshaikh/dna-system.git`). Overall, it is a slight variation of the established error functions from the MESA simulator [3]. There are possibilities to adapt the functions, and to equip them with other intermediate error functions (see `SuperBasicRules.java`). The implementation in `BasicDNARules.java` provides the error function of CBBs, InfoDNA, and oligos. Finally, `DistanceCoder.java` contains the score function for Equation 13 for the segmentation of long oligos.

7 Fisher-Yates Permutations

Our encoding algorithms compute permutations for a given DNA sequence and choose the best permutation based on the error function described in the section above. We implement the classical Fisher-Yates shuffle method to generate uniform permutations [4]. This method utilizes a random numbers generator (RNG), from which random integers are sampled. These integers are then used as index pairs to swap.

The quality of the RNG significantly affects the quality of the obtained permutation. In particular, poor-quality RNGs often produce numbers that are biased and easily predictable [5]. Therefore, we use *Ranlux* [6], a robust and high-quality RNG for sampling the index pairs. Moreover, Ranlux offers four different quality levels, and we use the highest quality level in our implementation.

8 Data Packing

We use data packing to insert (pack) data objects into a byte stream. For example, packing the string "3.141592653589793" byte-wise in an empty byte stream results in 17 bytes. However, this string can be represented by an 8-byte floating-point number. Hence, we pack values of a relational table into a byte stream by specified data types. We implement `Packer.java` that can pack any of the following data types:

- `BYTE`: 1 byte integer.
- `SHORT`: 2 bytes integer.
- `INT`: 4 bytes integer.
- `LONG`: 8 bytes integer.
- `FLOAT`: 4 bytes floating point number.
- `DOUBLE`: 8 bytes floating point number.
- `STRING_8`: String with up to 255 characters.

- `STRING_16`: String with up to 65535 characters.

Numerical values that do not fit into these data types are either packed as `STRING_8` or `STRING_16`. Data objects with sizes that exceed the latter cannot be packed with our packer. However, we could add additional data types to support larger data objects.

9 DNA Packing

DNA packing is a method to encode integers as DNA. For example, converting the 32-bit integer 5 to binary yields 32 bits and encoding them to DNA by mapping every two consecutive bits to the respective base results in 16 bases. However, this can be done more efficiently. We implement `DNAPacker.java` that encodes the integer 5 to just three bases. We support the following integer types:

- `HALF_BYTE`: 4 bit integer.
- `BYTE`: 8 bit integer.
- `SHORT`: 16 bit integer.
- `INT_31`: 31 bit integer.

To encode the integer 5 to DNA, we represent it as 4 bits (data type `HALF_BYTE`), and every two consecutive bits are mapped to a quaternary digit. Then, we map the two obtained quaternary digits to DNA by the same rules known from `RotatingQuattro` in Table 1. We use a prefix base to mark the data type. For example, finding an `A` corresponds to the data type `HALF_BYTE` that stores 4-bit integers. Hence, the following two bases (corresponding to two quaternary digits) represent a 4-bit integer. New data types can be easily added if an integer is too large to be packed in the existing types. However, we did not need to pack larger integers than 31 bits.

10 Delta+Range Compression

This method is used to compress the lists generated by encoding non-unique attributes. We combine delta and range compression into Delta+Range for integer values. The delta compression calculates the difference between two subsequent integers, and range compression compresses these deltas into ranges. This method requires the input integers to be distinct and sorted in ascending order, and is implemented in `DeltaCoder.java`.

Example

Let the input integer sequence be $(5, 6, 7, 8, 15)$. First, the deltas d_i are computed as $d_1 = 6 - 5 = 1$, $d_2 = 7 - 6 = 1$, $d_3 = 8 - 7 = 1$, and $d_4 = 15 - 8 = 7$. The first number in the sequence is the starting bias b_0 and set to $b_0 = 5$. Next, we generate the string $b_0; d_1; d_2; d_3; d_4 = 5; 1; 1; 1; 7$. After that, we compress

the consecutive deltas that equal 1 into ranges, i.e., we replace these deltas with their count to $5, 3; 7$. The comma after b_0 indicates that the next number represents a range's size, not a delta value. Since a range cannot be empty, i.e., the size must be greater than 1, we store the range's size minus one as $5, 2; 7$. To represent the next integer (that is not a range), we append a semicolon to indicate the previous range's ending and follow by appending $d_4 - 1$ (because $d_4 > 0$). The final compressed output is given as $5, 2; 6$.

11 Supplementary Figures

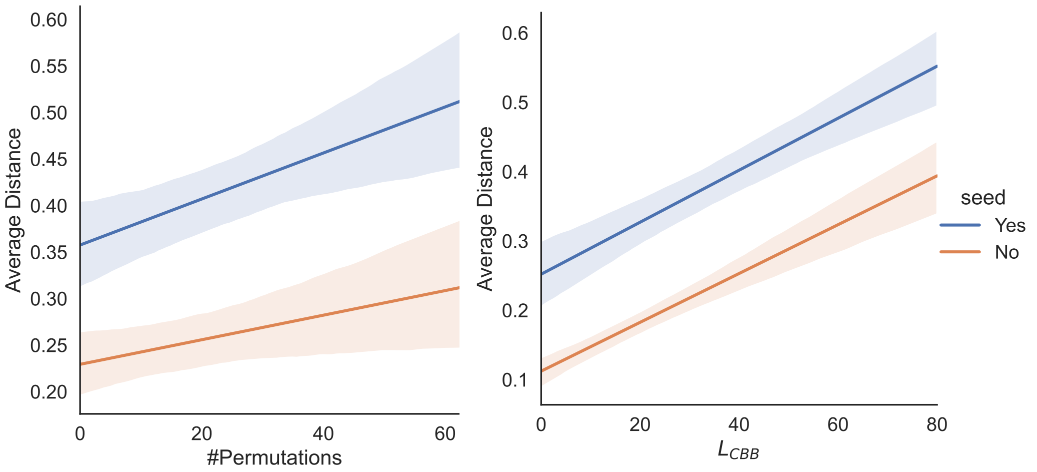

(a) (b)

Supplementary Figure 1: Shows the difference of the average distance of 1 million CBBs {`Id: 0,..., 999999`}, when we use the *seed* according to Equation (2) in the main manuscript versus when we do not use *seed* to skew the permutation's offset. The results are depicted as linear regression lines, where the lighter colors refer to the confidence intervals in size of the standard deviation. In **(a)** the difference is shown by varying the number of permutations and in **(b)** we depict the difference by varying L_{CBB}. We averaged the results over every DNA converters as there was no significant difference using a specific DNA converter.

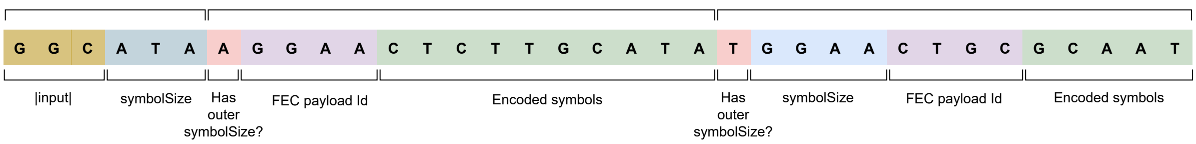

Supplementary Figure 2: The structure of a DNA strand generated by RQ.

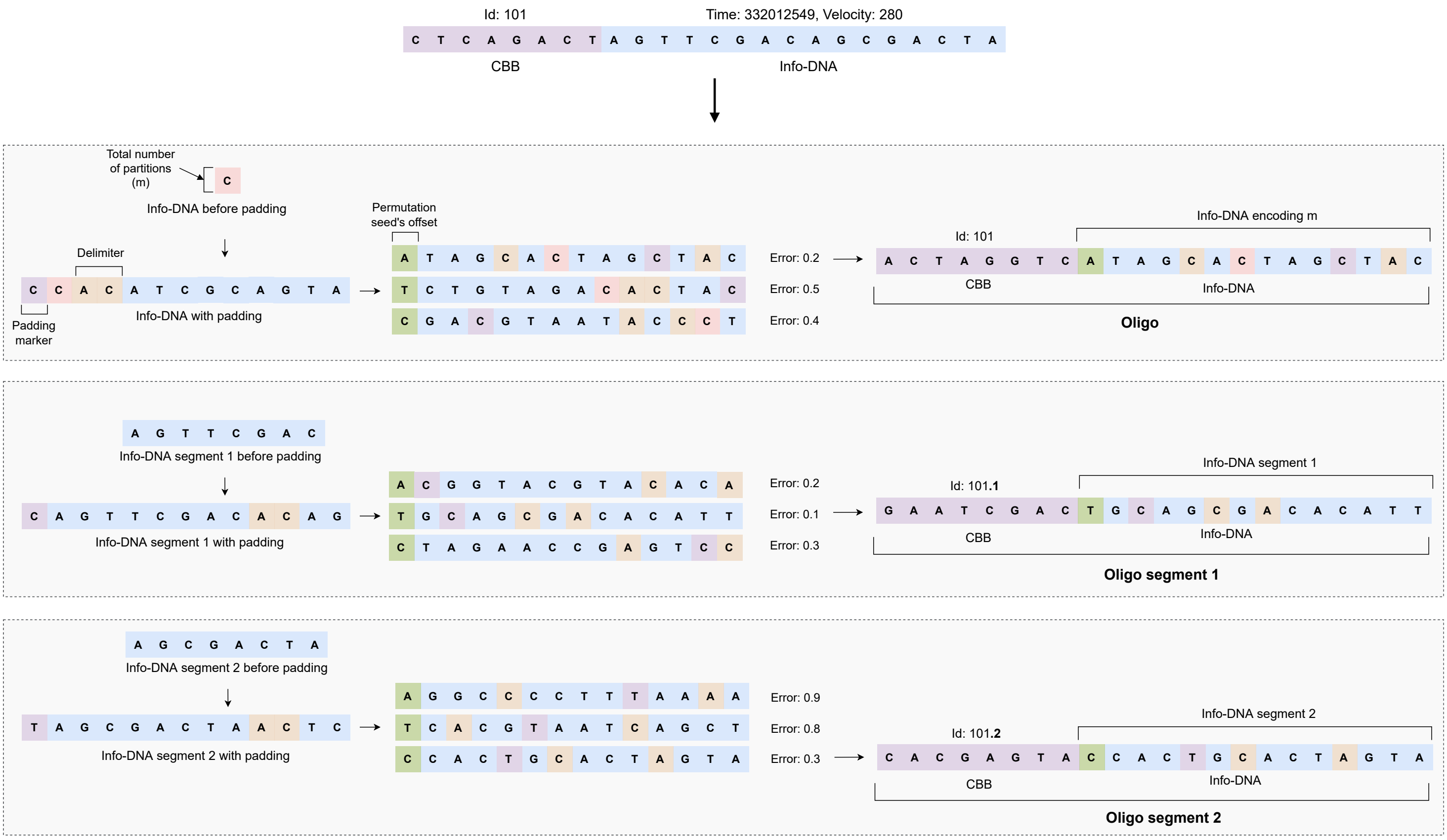

Supplementary Figure 3: An example for a given oligo that is segmented into three new oligos.

12 Supplementary Tables

#	[-2]	[-1]	Digit	Base
1	_	_	0	A
2	_	_	1	C
3	_	_	2	T
4	_	_	3	G
5	_	A	0	A
6	_	A	1	C
7	_	A	2	G
8	_	A	3	T
9	_	T	0	T
10	_	T	1	A
11	_	T	2	C
12	_	T	3	G
13	_	C	0	T
14	_	C	1	A
15	_	C	2	C
16	_	C	3	G
17	_	G	0	G
18	_	G	1	T
19	_	G	2	A
20	_	G	3	C
21	A	A	0	T
22	A	A	1	G
23	A	A	2	A
24	A	A	3	C
25	A	C	0	C
26	A	C	1	G
27	A	C	2	A
28	A	C	3	T
29	A	G	0	G
30	A	G	1	C
31	A	G	2	T
32	A	G	3	A
33	A	T	0	T
34	A	T	1	G
35	A	T	2	A
36	A	T	3	C
37	T	A	0	C
38	T	A	1	T
39	T	A	2	G
40	T	A	3	A
41	T	C	0	G
42	T	C	1	A
43	T	C	2	T
44	T	C	3	C
45	T	G	0	C
46	T	G	1	T
47	T	G	2	A
48	T	G	3	G
49	T	T	0	C
50	T	T	1	T
51	T	T	2	G
52	T	T	3	A
53	C	A	0	A
54	C	A	1	C
55	C	A	2	T
56	C	A	3	G
57	C	C	0	A
58	C	C	1	T
59	C	C	2	C
60	C	C	3	G
61	C	G	0	A
62	C	G	1	G
63	C	G	2	C
64	C	G	3	T
65	C	T	0	A
66	C	T	1	C
67	C	T	2	T
68	C	T	3	G
69	G	A	0	G
70	G	A	1	A
71	G	A	2	C
72	G	A	3	T
73	G	C	0	T
74	G	C	1	C
75	G	C	2	G
76	G	C	3	A
77	G	G	0	T
78	G	G	1	A
79	G	G	2	G
80	G	G	3	C
81	G	T	0	G
82	G	T	1	A
83	G	T	2	C
84	G	T	3	T

Supplementary Table 1: Mapping rules for `RotatingQuattro`.

#	[-1]	Digit	Base
1	A or _	0	C
2	A or _	1	G
3	A or _	2	T
4	C	0	G
5	C	1	T
6	C	2	A
7	G	0	T
8	G	1	A
9	G	2	C
10	T	0	A
11	T	1	C
12	T	2	G

Supplementary Table 2: Mapping rules for `RotatingTre`.

References

[1] Nick Goldman, Paul Bertone, Siyuan Chen, Christophe Dessimoz, Emily M LeProust, Botond Sipos, and Ewan Birney. Towards practical, high-capacity, low-maintenance information storage in synthesized dna. *Nature*, 494(7435):77–80, 2013.

[2] Alex El-Shaikh, Marius Welzel, Dominik Heider, and Bernhard Seeger. High-scale random access on DNA storage systems. *NAR Genomics and Bioinformatics*, 4(1), 01 2022. lqab126.

[3] Michael Schwarz, Marius Welzel, Tolganay Kabdullayeva, Anke Becker, Bernd Freisleben, and Dominik Heider. Mesa: automated assessment of synthetic dna fragments and simulation of dna synthesis, storage, sequencing and pcr errors. *Bioinformatics*, 36(11):3322–3326, 2020.

[4] Richard Durstenfeld. Algorithm 235: random permutation. *Communications of the ACM*, 7(7):420, 1964.

[5] Frederick James and Lorenzo Moneta. Review of high-quality random number generators. *Computing and Software for Big Science*, 4(1):1–12, 2020.

[6] Fred James. Ranlux: A fortran implementation of the high-quality pseudo-random number generator of lüscher. *Computer Physics Communications*, 79(1):111–114, 1994.
